# Development and assessment of a risk prediction model for moderate-to-severe obstructive sleep apnea

**DOI:** 10.3389/fnins.2022.936946

**Published:** 2022-08-05

**Authors:** Xiangru Yan, Liying Wang, Chunguang Liang, Huiying Zhang, Ying Zhao, Hui Zhang, Haitao Yu, Jinna Di

**Affiliations:** ^1^Department of Nursing, Jinzhou Medical University, Jinzhou, China; ^2^Sleep Monitoring Center, The First Hospital of Jinzhou Medical University, Jinzhou, China; ^3^Respiratory Medicine, The Third Hospital of Jinzhou Medical University, Jinzhou, China

**Keywords:** moderate-to-severe OSA, prediction model, nomogram, SBQ, NoSAS

## Abstract

**Background:**

OSA is an independent risk factor for several systemic diseases. Compared with mild OSA, patients with moderate-to-severe OSA have more severe impairment in the function of all organs of the body. Due to the current limited medical condition, not every patient can be diagnosed and treated in time. To enable timely screening of patients with moderate-to-severe OSA, we selected easily accessible variables to establish a risk prediction model.

**Method:**

We collected 492 patients who had polysomnography (PSG), and divided them into the disease-free mild OSA group (control group), and the moderate-to-severe OSA group according to the PSG results. Variables entering the model were identified by random forest plots, univariate analysis, multicollinearity test, and binary logistic regression method. Nomogram were created based on the binary logistic results, and the area under the ROC curve was used to evaluate the discriminative properties of the nomogram model. Bootstrap method was used to internally validate the nomogram model, and calibration curves were plotted after 1,000 replicate sampling of the original data, and the accuracy of the model was evaluated using the Hosmer-Lemeshow goodness-of-fit test. Finally, we performed decision curve analysis (DCA) of nomogram model, STOP-Bang questionnaire (SBQ), and NoSAS score to assess clinical utility.

**Results:**

There are 6 variables entering the final prediction model, namely BMI, Hypertension, Morning dry mouth, Suffocating awake at night, Witnessed apnea, and ESS total score. The AUC of this prediction model was 0.976 (95% CI: 0.962–0.990). Hosmer-Lemeshow goodness-of-fit test χ^2^ = 3.3222 (*P* = 0.1899 > 0.05), and the calibration curve was in general agreement with the ideal curve. The model has good consistency in predicting the actual occurrence of moderate-to-severe risk, and has good prediction accuracy. The DCA shows that the net benefit of the nomogram model is higher than that of SBQ and NoSAS, with has good clinical utility.

**Conclusion:**

The prediction model obtained in this study has good predictive power for moderate-to-severe OSA and is superior to other prediction models and questionnaires. It can be applied to the community population for screening and to the clinic for prioritization of treatment.

## Introduction

Obstructive sleep apnea (OSA) is a sleep disorder in which repeated collapsed obstruction of the upper airway during sleep with hypoventilation and decreased blood oxygenation leads to multisystem lesions and injury (Senaratna et al., 2017). Respiratory obstruction leads to arousal of the brain, sympathetic activation, and decreased oxygen saturation, and recurrent upper airway obstruction during sleep can lead to sleep fragmentation and non-restorative sleep. Therefore, people with OSA may have symptoms such as fatigue, excessive daytime sleepiness, or morning headaches, or some people may not have these symptoms. However, there is no denying that OSA is subtly damaging our body systems and organs.

OSA is classified into three levels: mild, moderate, and severe. According to the American Academy of Sleep Medicine (AASM) definition of OSA (Sateia, 2014; Kapur et al., 2017), the apnea hypoventilation index (AHI) is used to define the severity grading of OSA, 5 ≤ AHI < 15/h for mild OSA, 15 ≤ AHI < 30/h for moderate OSA, and AHI ≥ 30/h for severe OSA.

Seventeen studies in 16 countries provided reliable data on the prevalence of OSA, with an estimated 936 million adults aged 30–69 years worldwide suffering from mild-to-severe OSA and 425 million adults aged 30–69 years suffering from moderate-to-severe OSA (Benjafield et al., 2019). The largest number of people affected are in China, followed by the United States, Brazil, and India (Benjafield et al., 2019). About one in five adults has at least mild OSA, and one in fifteen has moderate or severe OSA (Somers et al., 2008). However, more than 85% of people with clinically significant and treatable OSA have never been diagnosed (Young et al., 1997; Kapur et al., 2002; Tufik et al., 2010).

Polysomnography (PSG) is the gold standard for diagnosing OSA. The device assesses the underlying cause of sleep disorders by monitoring the subject’s EEG, EOG, EMG, pulse oximetry, routine ECG, chest, and abdominal airflow movements, and lying position during sleep (Rundo and Downey, 2019). Sleep staging is determined by information from EEG, EOG, and EMG (Basunia et al., 2016). There are two types of sleep, namely non-rapid eye movement (NREM) sleep and rapid eye movement (REM). NREM sleep is divided into three sub-stages: N1, N2, and N3. Sleep stages usually begin with a shorter NREM stage 1 (N1), followed by stage 2 (N2), then stage 3 (N3), and finally REM (Basunia et al., 2016). A cyclic EEG alteration occurs during NREM, namely the cyclic alternating pattern (CAP), which reflects the microstructure of sleep and has a crucial role in establishing and maintaining sleep integrity (Terzano and Parrino, 2000; Smerieri et al., 2007). The CAP represents an adaptive state of persistent arousal instability that oscillates between higher levels of arousal and activation (stage A) and lower levels of arousal and deactivation (stage B) (Parrino et al., 2012; Gnoni et al., 2021). CAP and arousal are fundamental mechanisms of sleep regulation, with subtype A1 contributing to the accumulation and consolidation of deep slow-wave sleep (SWS), while subtypes A2 and A3 attenuate sleep and lead to episodes of REM sleep or arousal (Terzano et al., 2005). Studies have shown that CAP subtype A1 predominates in the mild OSA, while CAP subtypes A2 and A3 predominate in patients with moderate-to-severe OSA (Gnoni et al., 2021). In patients with mild OSA, CAP A1 subtypes may enhance sleep continuity, whereas, in moderate-to-severe OSA, there may be a loss of compensation of these sleep stabilization mechanisms and more invasive CAP fluctuations disrupting sleep circuits (Gnoni et al., 2021). Thus, compared to patients with mild OSA, patients with moderate-to-severe OSA constantly experience sleep fragmentation, producing physical symptoms such as drowsiness and fatigue, as well as psychological symptoms such as stress (Bardwell et al., 2000; Santos et al., 2017).

OSA is an independent risk factor for a variety of systemic diseases. Compared to mild OSA, moderate-to-severe OSA can cause more severe damage to various organs in the body. Cardiovascular disease is the most serious complication of OSA (Guilleminault et al., 1976), while hypertension is one of the clearest cardiac risk factors associated with OSA. Muxfeldt et al. (2014) analyzed 422 patients with intractable hypertension and found an overall prevalence of 82.2% and 55.5% for OSA and moderate-to-severe OSA, respectively, with a preponderance of non-dipping hypertension in patients of moderate-to-severe OSA (Gonzaga et al., 2015). On the other hand, there is sufficient evidence that moderate and severe OSA is associated with decreased ventricular function and increased atrial volumes, resulting in a high prevalence of chronic heart failure and atrial fibrillation in moderate-to-severe OSA patients (Sascău et al., 2018). In the cerebrovascular system, it has been reported that the more severe the OSA, the higher the risk of cerebrovascular disease, and there is a strong relationship between OSA and stroke (Yaggi et al., 2005; Lin et al., 2018). OSA is associated with early neurological deterioration, increased stroke recurrence, prolonged hospitalization, and decreased functional recovery, and adverse neurological outcomes are directly related to the severity of OSA (Yaggi et al., 2005; Yan-fang and Yu-ping, 2009; Loke et al., 2012). 57% of patients with chronic stroke have undiagnosed moderate-to-severe OSA (Gottlieb et al., 2021). In addition, it is worth noting that patients with moderate-to-severe OSA have a higher incidence of asymptomatic cerebrovascular disease than those with less severe OSA (Nishibayashi et al., 2008). OSA is also affecting the metabolism of endocrine hormone levels, and patients with OSA are at a higher risk of developing certain endocrine and metabolic diseases (Akset et al., 2022). The relationship between endocrine disorders and OSA is complex and bidirectional. Several endocrine disorders are risk factors for OSA. Compared to the general population, patients with obesity, hypothyroidism, acromegaly, Cushing’s syndrome, and type 1 and type 2 diabetes have an increased prevalence of OSA (Brúsik et al., 2016; Bruyneel et al., 2022). The prevalence of insulin resistance is also higher in non-diabetic patients with moderate-to-severe OSA (Michalek-Zrabkowska et al., 2021). A meta-analysis showed that OSA increased the risk of early kidney injury and that patients with moderate-to-severe OSA combined with hypertension and/or diabetes had significantly reduced glomerular filtration rate with more severe kidney injury (Liu et al., 2021). Meanwhile, moderate-to-severe OSA is a common cause of insomnia in patients (Hein et al., 2017).

Lazazzera et al. (2021) have designed a sleep monitoring platform that detects apnea and hypoventilation with a correct rate of up to 75.1%. At night patients need to wear smart gloves for signal acquisition and open a smartphone application where the information is transmitted to a remote server for cloud computing to estimate the status of sleep, breathing and heart rate during the night. Compared to other monitoring devices, this platform is less invasive and simpler. However, the platform requires the purchase of smart gloves for measurement and cannot distinguish the severity of OSA, so we believe it is not suitable for primary screening and can be used for post-treatment outcome evaluation. In patient interviews, it was found that most patients were not aware that they had OSA and considered it expensive to treat and examine, while on the other hand, OSA was not considered to be a health hazard and was even mistaken for a sign of good sleep. In recent years, tremendous efforts are being made to diagnose individuals with OSA, but data show that even in developed countries, most patients with OSA remain undiagnosed and untreated. In developing countries, OSA is poorly understood, often without diagnosis and treatment, and not adjusted to the lack of medical resources (Jaiswal et al., 2017). In China, the largest developing country, diagnosis, and treatment for OSA are also only carried out in tertiary hospitals. As people become more concerned about their health, awareness of OSA is slowly increasing, but due to the scarcity of monitoring equipment, PSG is often in short supply. Studies in China have shown that approximately 80% of males and 90% of females with moderate-to-severe disease do not receive a clinical diagnosis. The 5-year mortality rate for untreated OSA patients can be 11–13% (Young et al., 1993). The admission of patients with less severe OSA to the sleep monitoring unit further increases the strain on available resources. Given the dangers of moderate-to-severe OSA, it is imperative that patients with moderate-to-severe OSA are screened for PSG monitoring and treated as soon as possible, o realize the maximum utilization of resources.

However, there is no screening tool specifically for moderate-to-severe OSA. Therefore, we aim to build a predictive model that screens for moderate to severe OSA at no cost. We conducted a survey in the northeastern region of China, which has the highest prevalence of OSA(as shown in a meta-analysis of OSA prevalence by Chinese scholars in 2021) and developed a prediction model specifically for screening moderate-to-severe OSA based on risk factors and clinical symptoms of OSA patients. After reviewing the data, we included gender, age, BMI, neck circumference (NC), neck height ratio (NHR), history of hypertension, morning headache, Morning dry mouth, suffocating awake at night, witness respiratory pause, and ESS total score as predictor variables to develop the model, and compared the developed model with the current widely used tool (SBQ, NoSAS score) to evaluate its clinical effects.

## Materials and methods

### Study subjects

Patients who consulted the sleep monitoring centers of the First and Third Hospitals of Jinzhou Medical University and underwent PSG from 2017 to 2021 were included in this study. The study was approved by the Ethics Committee of Jinzhou Medical University (LLSC2020008) and was following the 1964 Declaration of Helsinki and subsequent amendments, and informed consent was obtained from each study subject before the study.

Inclusion criteria: (1) age ≥ 18 years; (2) have not been diagnosed with OSA. Exclusion criteria: (1) patients with severe cardiopulmonary disease and severe sleep disorders; (2) patients with uncontrolled mental illness; (3) patients with neuromuscular disease and a history of stroke. A total of 492 study subjects were included in this study.

### Predictor variables and outcome indicators

The study used easily measured general information and basic symptoms of patients as predictors, including gender, age, BMI, NC, NHR, history of hypertension, morning headache, morning dry mouth, suffocating awake at night, witness apnea, and ESS total score. Because of the low awareness of OSA, many people are not aware that morning headache, witness apnea, suffocating awake at night, morning dry mouth, daytime sleepiness is caused by OSA, but they only consider it as an ordinary phenomenon and do not draw attention to it. Therefore, we included them as predictor variables, the outcome variable of OSA severity. On the other hand, we predicted moderate-to-severe OSA, where symptoms are more prominent compared to mild OSA, so morning headache, witness apnea, suffocating awake at night, morning dry mouth, daytime sleepiness would have more predictive value.

The researchers measured the net height, weight (in light clothing), and NC of the study subjects. The NC is measured just below the cricoid cartilage, at the level of the mid-cervical spine (Zen et al., 2012). The normal reference values for neck circumference were < 38 cm for males and < 35 cm for females (Yang et al., 2010). BMI is calculated as the weight divided by the square of the height. The NHR is calculated by dividing the NC by the height. The Epworth Sleepiness Scale (ESS) (Johns, 1991) assesses the degree of daytime sleepiness in study subjects, which has a total of 8 entries and a total score of 24. It was validated by Chinese scholars in 2002, and the Cronbach’s α was 0.81, with good reliability (Chen et al., 2002). In addition, we calculated participants’ Stop-Bang Questionnaire (SBQ) (Chung et al., 2008) and NoSAS (Marti-Soler et al., 2016) scores to compare with our derived prediction model. Relevant study (Yu et al., 2012) in China have shown that the SBQ can be used to assess patients with high-risk OSA. It is a simple and easy-to-use screening and prediction tool for moderate-to-severe OSA with high sensitivity and negative predictive value (NPV). It is appropriate that the subject of this study is moderate-to-severe OSA, which can be compared with our prediction model.

All subjects included in this study underwent full-night PSG and were monitored for more than 7 h. The patient’s sleep is first automatically analyzed by the ResMed system software and then all information is manually evaluated by a professional sleep technician. Information such as the disease status of the study subjects was determined from the PSG report. According to the definition of OSA by the AASM (Sateia, 2014; Kapur et al., 2017), the cut-off point was AHI ≥ 15/h, and the group was divided into a disease-free mild OSA group (control group, AHI < 15/h) and a moderate-to-severe OSA group (AHI ≥ 15/h).

### Statistical methods

Statistical analysis of the data was performed using statistical analysis and graphing software with IBM SPSS 25.0, R 4.1.1, and GraphPad Prism 8.

### Variable description

The normality of continuous variables was assessed using the Kolmogorov-Smirnov goodness-of-fit test (Massey, 1951). Those that conformed to a normal distribution used the *t*-test, described as mean ± standard deviation; conversely, the Mann-Whitney *U*-test, described as median (interquartile spacing); and categorical variables used the chi-square test, described as frequency (percentage).

### Variable screen

First, the random forest model was used to determine the importance of NC and NHR and to select the appropriate risk factors. The importance of variables is calculated by using Gini Importance, which is used to determine the nodes of the individual decision trees, to generate Mean Decrease in Impurity (MDI) (Sydney, 2019). Each predictor variable used to create the random forest model has a resulting MDI value, which is used to rank variable importance to the model. Higher Mean Decrease in Gini indicates higher variable importance (Sydney, 2019). The selected predictive factors were used as independent variables one by one in the univariate analysis, and the variables with *p* < 0.05 were included in the diagnosis of multicollinearity to determine whether there was multicollinearity among the variables. The larger the value of variance inflation factor (VIF), the stronger the degree of multicollinearity among the variables, and it was generally considered that factors with VIF > 5 had strong collinearity among them (Chatterjee et al., 2000). The selected independent variables were included in a binary logistic regression analysis using forward stepwise regression to obtain the predictive factors for moderate-to-severe OSA and the strength of their associations.

### Nomogram and evaluation

The variables screened in the binary logistic regression analysis were used as the final predictors to create a nomogram predicting the probability of moderate-to-severe OSA. We evaluated the model in terms of its discrimination, accuracy and clinical usefulness. Mature clinical prediction models require three necessary stages: construction, internal validation, and external validation. Internal validation is the evaluation of the model using data from sources similar to those used to construct the prediction model, while external validation is the evaluation of the model with data that are spatially (multicenter) different from those used for model construction. Our data are from the population of Liaoning region, and if the internal validation is performed the population is also the same population, so we believe that dividing the data into training and validation sets is not necessary in our study. We perform internal validation on the original data using Bootstrap method with 1,000 repetitions of sampling.

#### Discrimination

The area under the curve (AUC) was used to assess the differentiation of the nomogram model, and the sensitivity, specificity, positive predictive value (PPV), and NPV of the model were calculated to evaluate the discriminatory capability of the model.

#### Accuracy

The Bootstrap method was used for internal validation of the nomogram model, and the calibration curve was plotted after 1,000 repetitions of the original data, while the consistency of the model was judged using the Hosmer-Leme show test, and *P* > 0.05 was considered as a good degree of predictive conformity of the model (Hosmer and Lemeshow, 2000).

#### Clinical utility

Decision curve analysis (DCA) is a metric to assess the predictive value and clinical utility of the model, and differentiating between patients at high and low risk of occurrence is the primary purpose of the model. Finally, we performed DCA of nomogram model, SBQ, and NoSAS to assess clinical utility. DCA estimates the net benefit of the prediction model based on the difference between the number of correctly and incorrectly predicted positive outcomes (Vickers and Holland, 2021).

**FIGURE 1:**
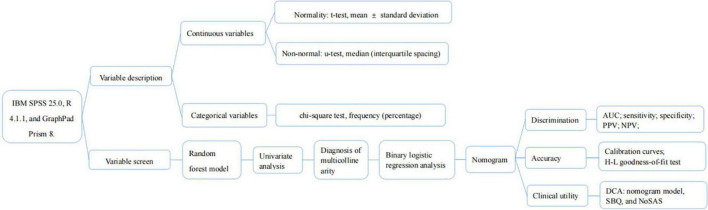
Flow chart of statistical process.

The flow chart of the statistical method is shown in Figure 1.

## Results

### Basic information about variables

**FIGURE 2:**
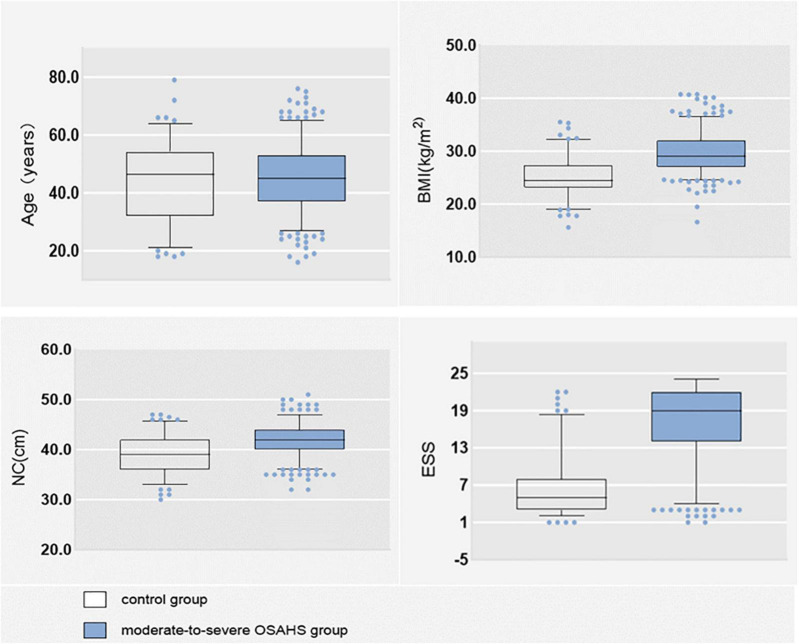
Box plot of age, BMI, NC, and ESS.

Of the 492 subjects, 366 (74.390%) had moderate-to-severe OSA and 126 (25.610%) had no disease and mild OSA. There were 375 (76.220%) males and 117 (23.780%) females; 347 (70.528%) with hypertension; 175 (35.569%) with morning headache; 346 (70.325%) with morning dry mouth; 337 (68.496%) with nocturnal awakening; and 345 (70.122%) with witnessed apnea. The mean NHR was 0.242. The data distributions of age BMI, NC, and total ESS score were expressed as medians, and their box plots are shown in Figure 2, and the specific values of each variable are shown in Table 1.

**TABLE 1:** Information about the study subjects and single-factor analysis.

Predictive factors	All subjects (*N* = 492)	Comparison of predictive factors between the two groups [M (P25, P75)/Mean ± SD]			
		
		Disease-free mild OSA group (*n* = 126)	Moderate-to-severe OSA group (*n* = 366)	Z/t/χ^2^	*P*
Gender				28.583	0.003
Male	375 (76.220%)	74	301		
Female	117 (23.780%)	52	65		
Age (years)	46.0 (36.0, 53.0)	46.5 (32.0, 54.25)	46.0 (37.0, 53)	−0.944	0.345
BMI (kg/m^2^)	28.374 (25.543, 31.157)	24.405 (23.146, 27.341)	29.055 (27.051, 32.010)	−10.405	0.000
NC (cm)	42 (39, 44)	39 (36, 42)	42 (40, 44)		
NHR (cm)	0.242 ± 0.019	0.230 ± 0.019	0.246 ± 0.018	−8.612	0.000
Hypertension				109.891	0.000
Yes	347 (70.528%)	23 (4.675%)	324 (65.854%)		
No	145 (29.472%)	103 (20.9355%)	42 (8.537%)		
Morning headache				82.804	0.000
Yes	175 (35.569%)	25 (5.081%)	150 (30.488%)		
No	317 (64.431%)	101 (20.528%)	216 (43.902%)		
Morning dry mouth				123.148	0.000
Yes	346 (70.325%)	27 (5.488%)	319 (64.837%)		
No	146 (29.675%)	99 (20.122%)	47 (9.553%)		
Suffocating awake at night				131.122	0.001
Yes	337 (68.496%)	27 (5.488%)	315 (64.024%)		
No	155 (31.504%)	99 (20.122%)	51 (10.366%)		
Witness apnea				123.148	0.000
Yes	345 (70.122%)	23 (4.675%)	322 (65.447%)		
No	147 (29.878%)	103 (20.935%)	44 (8.943%)		
ESS total score	17 (7, 21)	5 (3, 5)	20 (15, 22)	−12.997	0.000

### Screening variables

#### Random forest model screening variables

**FIGURE 3:**
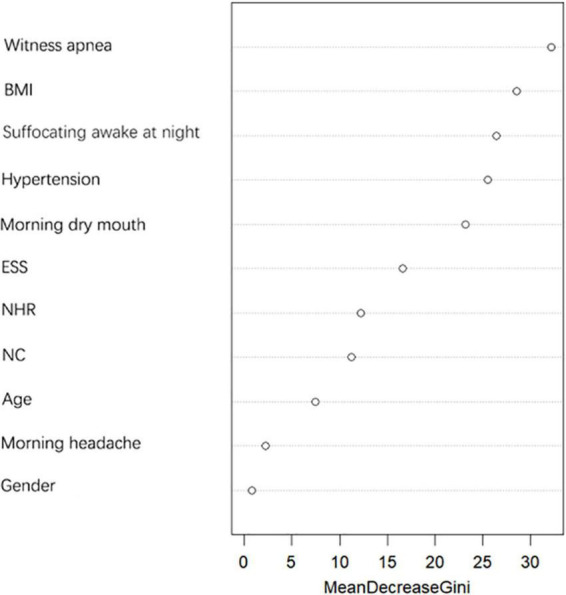
Variable importance Plot — MeanDecreaseGini.

The importance of predictors on the dependent variable was determined by the random forest model, and the results are shown in Figure 3. NHR has better predictive power for moderate-to-severe OSA compared to NC, therefore, NC was excluded and NHR was included as a predictor in this study.

#### Univariate analysis and multicollinearity test

Univariate analysis of predictors between the disease-free mild OSA group and moderate-to-severe OSA group showed statistically significant differences (*P* < 0.05) between the nine factors of gender, BMI, NHR, hypertension, morning headache, morning dry mouth, suffocating awake at night, witness apnea, and ESS total score, while there was no statistically significant difference between the two groups in terms of age. Details are shown in Table 1 the nine statistically significant predictive factors derived above were tested for multiple covariances. The VIF of the included predictive factors was < 5, so there was no covariance. The predictors were also assigned values for binary logistic regression analysis, and the covariance test results and assignments are shown in Table 2.

**TABLE 2:** Multicollinearity test of predictors and the way to assign values.

Risk factors	Tolerance	VIF	Assignment
Gender	0.881	1.135	“Male” = 1, “female” = 2
BMI (kg/m^2^)	0.583	1.715	Original value entry
NHR	0.616	1.624	Original value entry
Hypertension	0.527	1.898	“No” = 0, “yes” = 1
Morning headache	0.928	1.078	“No” = 0, “yes” = 2
Morning dry mouth	0.612	1.635	“No” = 0, “yes” = 1
Suffocating awake at night	0.533	1.877	“No” = 0, “yes” = 1
Witnessed apnea	0.517	1.935	“No” = 0, “yes” = 1
ESS total score	0.578	1.731	Original value entry

#### Binary logistic regression analysis

A binary logistic regression analysis was performed with whether the OSA was moderate-to-severe (“no” = 0, “yes” = 1) as the dependent variable and the nine statistically significant predictors mentioned above (gender, BMI, NHR, hypertension, morning headache, morning dry mouth, suffocating awake at night, witnessed apnea, and ESS total score) as the independent variables; the assignment table is shown in Table 2. The results of the binary logistic regression analysis showed independent predictors of BMI, hypertension, morning dry mouth, suffocating awake at night, witnessing apnea, and ESS total score for moderate-to-severe OSA, as detailed in Table 3.

**TABLE 3:** Results of binary logistic regression analysis.

Predictive factors	B	SE	Wals	*P*	OR	95% CI
BMI (kg/m^2^)	0.454	0.087	27.478	0.000	1.574	1.329–1.865
Hypertension	1.463	0.501	8.510	0.004	4.317	1.616–11.532
Morning dry mouth	1.911	0.493	15.054	0.000	6.759	2.574–17.748
Suffocating awake at night	2.080	0.522	15.893	0.000	8.004	2.879–22.254
Witnessed apnea	1.558	0.530	8.623	0.003	4.747	1.679–13.427
ESS total score	0.242	0.045	28.535	0.000	1.274	1.166–1.393
Constants	–18.238	2.948	38.266	0.000	0.000	

### Construct and evaluate the nomogram

**FIGURE 4:**
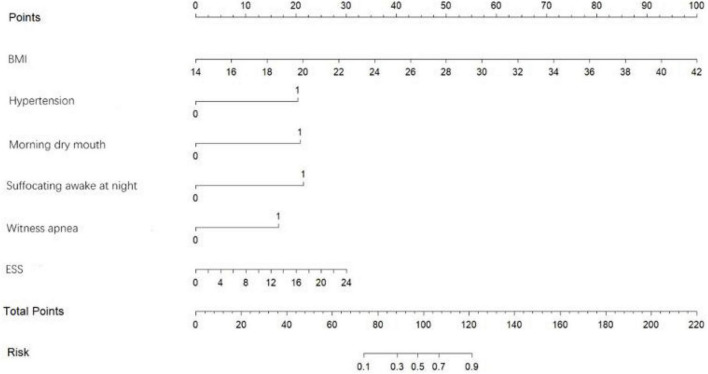
Predicting the risk of moderate-to-severe OSA in the nomogram.

Based on the predictor variables derived from the above binary logistic regression analysis, we created the nomogram shown in Figure 4. The scores corresponding to each patient’s six indicators of BMI, hypertension, morning dry mouth, suffocating awake at night, witnessing apnea, and ESS total score in the nomogram were summed to calculate the total score, and the risk of having moderate-to-severe OSA for that patient could be derived.

#### Discrimination

**FIGURE 5:**
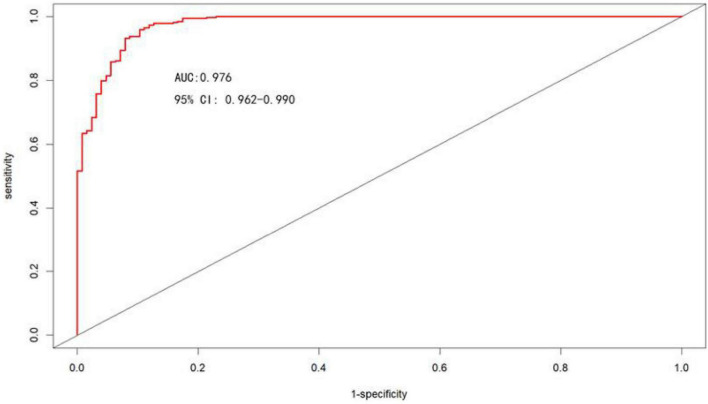
ROC curves for the nomogram model to predict the risk of moderate-to-severe OSA occurrence.

The area under the ROC curve was used to assess the discrimination of the nomogram model, as shown in Figure 5, the area under the ROC curve: AUC = 0.976 (95% CI: 0.962–0.990), the cut-off value was 0.620, and sensitivity, specificity, PPV, and NPV were 95.9%, 89.7%, 96.4%, and 88.3%, respectively, suggesting that the nomogram model has good discrimination ability.

#### Accuracy

**FIGURE 6:**
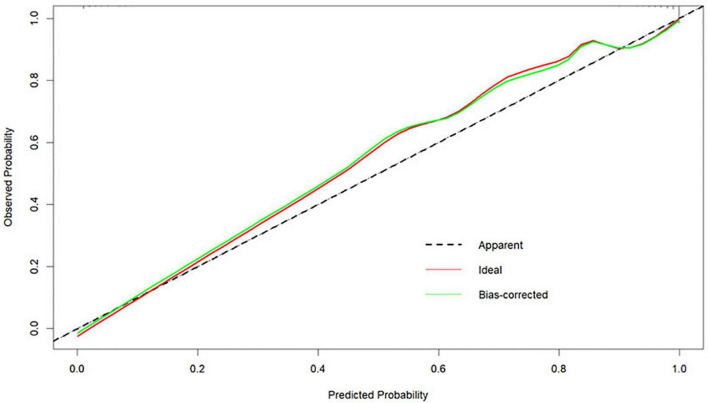
Calibration curves for the nomogram model predicting the risk of developing moderate-to-severe OSA.

The Bootstrap method was used to internally validate the nomogram model, and the calibration curve was plotted after the original data were repeatedly sampled 1,000 times (see Figure 6), while the Hosmer-Lemeshow goodness-of-fit test χ^2^ = 3.3222, *P* = 0.1899, and there was no statistically significant difference between the risk prediction value and the actual observation value, suggesting that the model predicts the actual occurrence of moderate-to-severe risk with good agreement, indicating that the model has good prediction accuracy.

#### Clinical utility

**FIGURE 7:**
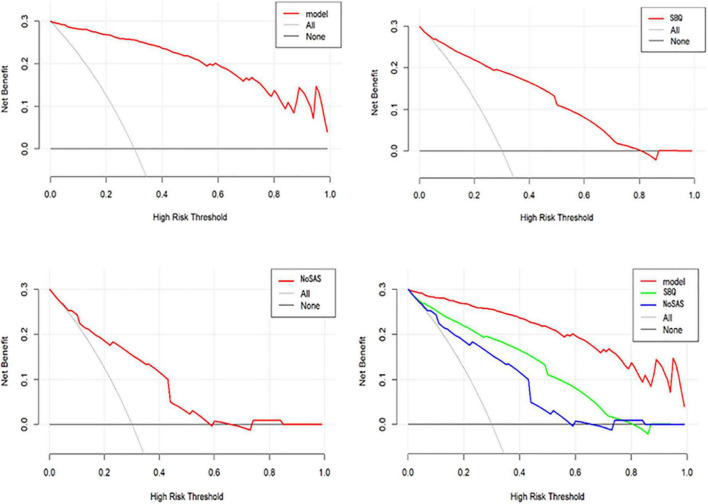
DCA of the nomogram model, SBQ, NoSAS.

**FIGURE 8:**
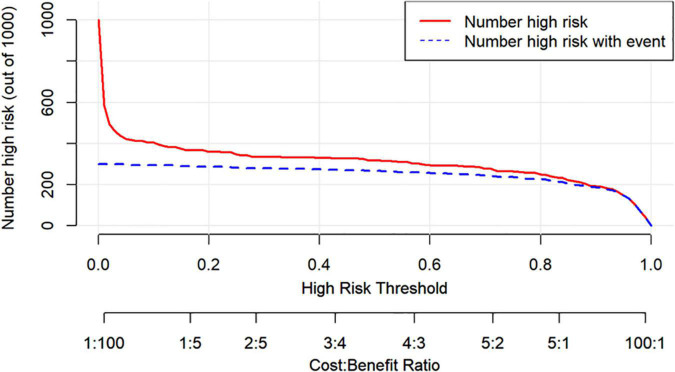
The clinical impact curves of the nomogram model.

Figure 7 shows the DCA for the nomogram model, SBQ, and NoSAS. The DCA has the threshold probability as the horizontal coordinate and the net benefit rate after subtracting the disadvantage as the vertical coordinate, and the closer the curve is to the upper right corner, the greater the net clinical benefit obtained using this prediction model. The graph shows that the net benefit of the nomogram model is higher than that of SBQ and NoSAS. Figure 8 shows the clinical impact curves of the nomogram model. The red curve (Numberhigh risk) represents the number of individuals classified as positive (high risk) by the nomogram model at each threshold probability; the blue curve (Number high risk with outcome) is the number of true positives at each threshold probability.

**FIGURE 9:**
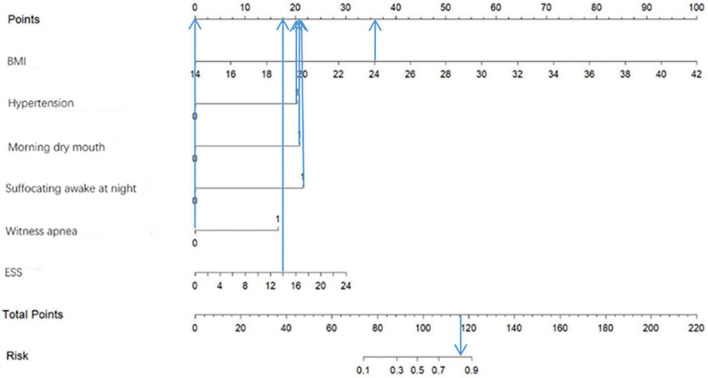
Example of nomogram.

Figure 9 shows an example of a nomogram for a patient. This patient has a BMI of 24, hypertension, dry mouth in the morning, no apnea in others, suffocating awakenings at night, and an ESS score of 14. According to the nomogram model, this patient has a total score of 116.5 and an approximately 84% probability of having moderate-to-severe OSA.

## Discussion

OSA is a recognized sleep disorder, and the number of PSG devices, the gold standard for diagnosis of OSA, is limited and often in short supply. Compared with mild OSA, moderate-to-severe OSA is more harmful to health. In order to achieve the maximum utilization of resources, this study selected simple and easy-to-measure general information and clinical symptoms of patients as predictors and established a prediction model for moderate-to-severe OSA. It helps to realize the difficult task of rapid screening of patients with moderate-to-severe OSA. In the following, we describe the selection of variables and the basic conditions of the model developed.

### Gender

OSA has long been considered to be associated with males, and many studies have shown a higher prevalence among males than females (Kapsimalis and Kryger, 2002). Data from sleep labs suggest that male risk of OSA is 5–6 times higher compared to female risk, while results from community studies suggest that male risk of OSA is only 2–3 times higher (Flemons, 2002; Daltro et al., 2006), so we included gender in this study to explore the relationship between gender and moderate-to-severe OSA. Our findings showed a statistical difference between genders in whether or not to have moderate-to-severe OSA (as shown in Table 1), but random forest plots showed that gender was a poor predictor of moderate-to-severe OSA (as shown in Figure 3) and ultimately did not enter into our prediction model (as shown in Figure 4). Possibly because of the relatively mild degree of disease and atypical symptoms in females with OSA, studies suggest that failure to identify gender-specific differences may lead to under-diagnosis or misdiagnosis of OSA in females (Kapsimalis and Kryger, 2002). There are many prediction models or screening questionnaires that include gender as a predictor (Chen et al., 2002; Zou et al., 2013; Marti-Soler et al., 2016; Tawaranurak et al., 2021; Song et al., 2022), which has a high potential to cause under-diagnosis in female patients. In our prediction model, gender is not included, which largely reduces the underdiagnosis rate in female patients with moderate-to-severe OSA.

### Age

Many studies have shown a strong correlation between age and OSA (Ancoli-Israel et al., 1991; Liu et al., 2022), as age increases, the muscles in the nasopharynx relax and tend to collapse. Kapsimalis and Kryger (2002) found that 61.2% of people aged < 55 years had OSA, 78% of people aged > 55 years had OSA, and the prevalence of OSA increased significantly after age > 60 years. In contrast, in our study, age was not statistically different between moderate-to-severe OSA and disease-free mild OSA group (*p* < 0.05), and the medians were similar between the two groups (as shown in Figure 2 box plots). The reason for this analysis may be related to our sample population, all of us were Chinese. It was shown that the Chinese have underlying craniofacial skeletal differences compared to Caucasians, with significantly smaller maxillae and mandibles, more severe mandibular retrusion, anterior lower incisors, and increased total and upper facial height, and steeper and shorter anterior cranial bases (Liu et al., 2000). We speculate that the natural cranial characteristics of the Chinese population may contribute to moderate-to-severe OSA, and that the correlation with age may be relatively low.

### BMI, neck circumference, and neck height ratio

Obesity is a recognized risk factor for OSA (Daltro et al., 2006). Studies have shown that for every 10% increase in body weight, the risk of the probability of developing moderate-to-severe OSA increases sixfold (Peppard et al., 2000). In our study, BMI was significantly higher in moderate-to-severe OSA than in controls (as shown in Figure 2). Each 1 unit increase in BMI was associated with a 1.574-fold increase in the probability of having moderate-to-severe OSA (as shown in Table 3). Related obesity studies have shown that obesity can lead to a decrease in lung volume, resulting in a decrease in pharyngeal diameter; on the other hand, obesity leads to a narrowing of the pharynx and fat deposition on the pharyngeal wall, which is strongly related to NC, and many scholars have included NC in studies of OSA, and the findings have found a correlation between NC and OSA (Simpson et al., 2010; Kawaguchi et al., 2011; Ahbab et al., 2013). There is also evidence that NHR is superior to NC when assessing upper body fat distribution in patients with OSA (Ho et al., 2016). And in our study, we compared the prediction performance of NC and NHR for moderate-to-severe OSA by random forest model, and the results showed that the prediction ability of NHR was better than that of NC (as shown in Figure 3), so we included the NHR in this study. Unfortunately, the NHR did not enter into final prediction model, which is different from the previous studies (Ho et al., 2016). However, we believe that BMI, NC, and NHR are all indicators for assessing body obesity, and it is sufficient to include one indicator in our model to measure body obesity.

### Hypertension

There is a strong association between OSA and hypertension, and OSA is a major risk factor for hypertension (Cai et al., 2016). In contrast to hypertension, awareness of OSA is low and a large number of people are unaware that hypertension is often caused by OSA, therefore we will select a history of hypertension as a predictor for the prediction model. In our study, 88.525% of patients with moderate-to-severe OSA had hypertension, which is slightly lower than the previous study by Wang et al. (2016) (94.41% prevalence of hypertension in moderate-to-severe OSA), which may be related to the sample size. Hypertension was a predictor of OSA, which is similar to many previous studies (Chung et al., 2008; Marti-Soler et al., 2016; Tawaranurak et al., 2021) and we will not elaborate too much on it, as detailed in Tables 1, 3.

### Morning headache

Sleep and headache have a complex interrelationship (Stark and Stark, 2015), morning headaches are common in habitual snorers, and a study by Chen et al. (2011) found OSA to be an independent predictor of headache. Morning headaches were found to be significantly higher in those with OSA compared to those without OSA, with the prevalence of sleep apnea headaches being 11.6% and 13.3% in those with moderate and severe OSA, respectively (Kristiansen et al., 2012). However, some studies have also found that OSA is not associated with headaches (Russell et al., 2014; Suzuki et al., 2015). In our study, morning headache was statistically different between moderate-to-severe OSA and disease-free mild OSA group (*p* < 0.05), but this variable did not enter into our prediction model. We reviewed previous screening models and questionnaires and almost none of them included morning headache as a variable. The specifics between moderate-to-severe OSA and disease-free mild OSA are yet to be confirmed by a large amount of data.

### Morning dry mouth

Dry mouth is a typical clinical symptom of OSA, and morning dry mouth increases the likelihood of OSA in patients. One study combined morning dry mouth with the SBQ, which could improve the sensitivity and specificity of the questionnaire (Zhang et al., 2021). Oksenberg et al. (2006) found that the prevalence of dry mouth upon waking increased with the severity of OSA. In our study, 70.325% reported dry mouth and the risk of moderate-to-severe OSA was 1.635 times higher in those who reported dry mouth than in those who had no disease or mild OSA (as shown in Tables 1, 3).

### Witnessed apnea and Suffocating awake at night

OSA, as the name implies, results in apnea, a condition that is highly likely to occur in patients with moderate-to-severe OSA. Because of the airway obstruction, the lack of oxygen causes the brain to stimulate microarousal or arousal, which in turn may cause the patient to wake up suffocating at night. The majority of patients who came to the clinic complained of suffocating awake at night, 70.122% of all our study subjects witnessed apnea and 68.496% experienced suffocating awake at night (as shown in Table 1), and both were statistically significant (*P* < 0.05) between moderate-to-severe OSA and control groups (as shown in Table 3).

### Epworth sleepiness scale total score

The most common symptom of OSA is excessive sleepiness, which is reported by only 15–50% of patients with OSA in the general population (Gottlieb and Punjabi, 2020). The ESS is a measure of daytime sleepiness. In the clinical evaluation of OSA, the ESS is often used as a tool to measure daytime sleepiness (Pouliot et al., 1997). ESS has important value in the assessment of patients with severe OSA (Lee et al., 2020), but it has also been shown that ESS should not be used in clinical settings for individual-level comparisons to determine whether to make a priority diagnosis (Lee et al., 2020). Therefore, in our study, ESS was combined with several variables to create a predictive model that can screen for moderate-to-severe OSA. As can already be seen in Figure 2, the ESS total score was significantly higher in patients with moderate-to-severe OSA than in controls and statistically different between the two groups (*p* < 0.05). However, a random forest plot and nomogram (Figures 3, 4) shows that the ESS total score has a moderate ability to predict moderate-to-severe OSA, similar to previous studies (Chiu et al., 2017).

### Prediction model

In conclusion, in our study, the model for predicting moderate-to-severe OSA had six variables, namely BMI, hypertension, morning dry mouth, suffocating awake at night, witnessing apnea, and ESS total score. We visualize the model and represent it as a nomogram. The ROC curve and Hosmer-Lemeshow goodness-of-fit test showed that the prediction model had good discrimination and calibration; AUC = 0.976 (95% CI: 0.962–0.990), sensitivity, and specificity of 95.9% and 89.7%, respectively, Hosmer-Lemeshow goodness-of-fit test χ^2^ = 3.3222 (*P* = 0.1899 > 0.05). In clinical application, the scores corresponding to the risk factors can be summed to obtain a total score, and the risk of having moderate-to-severe OSA can be judged by the total score. The predictive model with the greatest net benefit for any given probability threshold is the optimal model. The DCA derived from this study shows that outcome prediction using our final resulting nomogram has greater net benefit than the SBQ and NoSAS scores for screening patients with moderate-to-severe OSA.

The sensitivity and specificity for identifying OSA in the predictive model for screening OSA developed by Tawaranurak et al. (2021) were approximately 93% and 26%, respectively, with a lower specificity. Kushida et al. (1997) developed a morphological model using BMI, NC, and oral cavity measurements by 1997, with a sensitivity of 97.6% and a specificity of 100%. Some morphological variables were also included in our study, but unfortunately, only BMI entered our model. Xu et al. (2019) also developed a model to predict OSA based on anthropometric variables, with AUCs of 0.755 and 0.788 for predicting moderate and severe OSA, respectively. Xu et al. (2019) also mentioned in the limitations of their article that the model did not incorporate clinical symptoms and medical history. This regret was remedied in the model we developed by including both anthropometric variables as well as clinical features and medical histor, which is the reason for the high predictive power of our predictive model, which is inseparable from the combination of variables. Lin et al. (2019) developed a prediction model with AHI as the dependent variable, which was more complicated to calculate and predicted moderate-to-severe OSA with AUC = 0.816, and our model outperformed it and the model was simple and easy to calculate. In 2010, Taipei Medical University based on an artificial intelligence system a prediction model for screening moderate-to-severe OSA was developed, and the study showed that both weight and height showed no statistical significance in the model, and the genetic algorithm involved (chromosomal examination) was not applicable to the screening of the general population (Sun et al., 2011). Other than that, we did not identify predictive models dedicated to screening for moderate-to-severe OSA.

Several screening questionnaires have been widely used for OSA, such as the Berlin questionnaire (BQ), SBQ, STOP questionnaire, and ESS. Studies have shown that the SBQ is more accurate in detecting mild, moderate, and severe OSA compared to the BQ, STOP, and ESS (Chiu et al., 2017). SBQ has higher sensitivity than other tools but lower specificity than ESS, so ESS was included in our study to improve the specificity of our model (Chiu et al., 2017). NoSAS score is a new tool that has been widely used in recent years to screen for OSA. Many studies have shown that the NoSAS score is more accurate than previous tools (BQ, SBQ, etc.) (Marti-Soler et al., 2016). A Meta-analysis of NoSAS showed a combined sensitivity of 79.8%, a combined specificity of 58.2%, and the AUC was 0.77 (Chen et al., 2022). When evaluating moderate-to-severe OSA, the AUC was 0.746, the sensitivity was 68.2% and the specificity was 75.4% (Costa et al., 2020). Our predictive model still outperformed the NoSAS score compared to our model.

## Limitation

The model was developed based on the Chinese population and may not be applicable to other ethnic groups. In this study, internal validation was conducted due to limited sample size, and no validation group was established to validate this prediction model. It is hoped that the model will be externally validated in future studies by conducting multicenter studies to increase the sample size, and applying it to community populations for screening to assess the performance of the high model. The model developed in this study was based on the severity of OSA and the results of this study differed from previous studies, possibly due to different groupings, and future prediction models could attempt to link to AHI.

## Conclusion

The prediction model developed in this study included six variables, namely BMI, Hypertension, Morning dry mouth, Suffocating awake at night, Witnessed apnea, ESS total score, which had good predictive power for moderate-to-severe OSA and was superior to other prediction models and questionnaires. The model is simple, does not use invasive tests, and the selected predictors are all easily collected variables, which makes it easy to use and can be applied to both community populations for screening and clinical settings to prioritize treatment.

## Data availability statement

The original contributions presented in this study are included in the article/supplementary material, further inquiries can be directed to the corresponding author.

## Ethics statement

The studies involving human participants were reviewed and approved by the Ethics Committee of Jinzhou Medical University (LLSC2020008). The patients/participants provided their written informed consent to participate in this study. Written informed consent was obtained from the individual(s) for the publication of any potentially identifiable images or data included in this article.

## Author contributions

XY conducted the data analysis and wrote this article. CL, HuiZ, and HY directed the study. HuiyZ, LW, YZ, and JD collected the data for this study. All authors conceived to the study.
